# Creatine metabolism in patients with urea cycle disorders

**DOI:** 10.1016/j.ymgmr.2021.100791

**Published:** 2021-08-23

**Authors:** Filippo Ingoglia, Jean-Leon Chong, Marzia Pasquali, Nicola Longo

**Affiliations:** a Department of Pathology, University of Utah, Salt Lake City, UT 84108, USA; b Department of Pediatrics, University of Utah, Salt Lake City, UT 84108, USA; c ARUP Laboratories, 500 Chipeta Way, Salt Lake City, UT 84108, USA

**Keywords:** Arginine, Creatine, Guanidinoacetate, Creatine deficiency, Urea cycle defect, Arginase deficiency, AGAT, arginine glycine amidinotransferase, ASS, argininosuccinate synthase, ASL, argininosuccinate lyase, CT1, creatine transporter 1, GAA, guanidinoacetate, GAMT, guanidino acetate methyltransferase, NOS, nitric oxide synthase, ORNT1, ornithine transporter 1, OTC, ornithine transcarbamylase, *SLC6A8*, solute carrier family 6 member 8 gene, UCD, urea cycle disorders

## Abstract

The urea cycle generates arginine that is one of the major precursors for creatine biosynthesis. Here we evaluate levels of creatine and guanidinoacetate (the precursor in the synthesis of creatine) in plasma samples (n_s_ = 207) of patients (n_p_ = 73) with different types of urea cycle disorders (ornithine transcarbamylase deficiency (n_s_ = 22; n_p_ = 7), citrullinemia type 1 (n_s_ = 60; n_p_ = 22), argininosuccinic aciduria (n_s_ = 81; n_p_ = 31), arginase deficiency (n_s_ = 44; n_p_ = 13)). The concentration of plasma guanidinoacetate positively correlated (*p* < 0.001, R^2^ = 0.64) with levels of arginine, but not with glycine in all patients with urea cycle defects, rising to levels above normal in most samples (34 out of 44) of patients with arginase deficiency. In contrast to patients with guanidinoacetate methyltransferase deficiency (a disorder of creatine synthesis characterized by elevated guanidinoacetate concentrations), creatine levels were normal (32 out of 44) or above normal (12 out of 44) in samples from patients with arginase deficiency. Creatine levels correlated significantly, but poorly (*p* < 0.01, R^2^ = 0.1) with guanidinoacetate levels and, despite being overall in the normal range in patients with all other urea cycle disorders, were occasionally below normal in some patients with argininosuccinic acid synthase and lyase deficiency. Creatine levels positively correlated with levels of methionine (*p* < 0.001, R^2^ = 0.16), the donor of the methyl group for creatine synthesis. The direct correlation of arginine levels with guanidinoacetate in patients with urea cycle disorders explains the increased concentration of guanidino compounds in arginase deficiency. Low creatine levels in some patients with other urea cycle defects might be explained by low protein intake (creatine is naturally present in meat) and relative or absolute intracellular arginine deficiency.

## Graphical abstract

**Figure:**
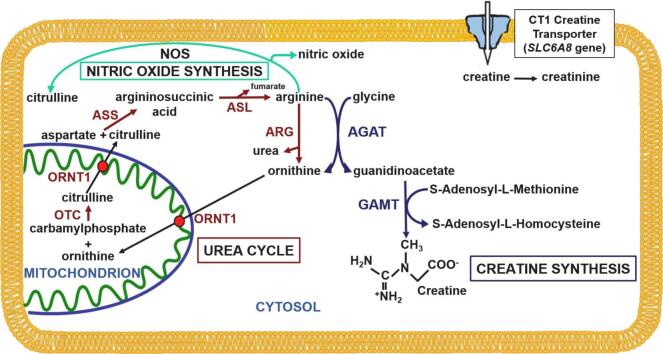

## 1 Introduction

**Fig. 1:**
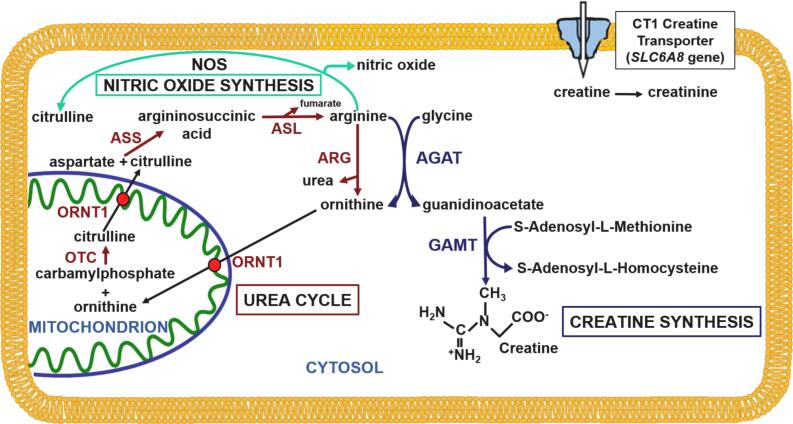
**Interaction between the urea cycle and creatine synthesis.** Arginine (a product of the urea cycle) is the key precursor of creatine. Ornithine, an intermediate of the urea cycle, is produced by the first reaction in the synthesis of creatine and, in excess, can inhibit creatine synthesis. Abbreviations: AGAT: arginine glycine amidino transferase, ARG: arginase, ASL: argininosuccinate lyase, ASS: argininosuccinate synthase, CT1: creatine transporter 1, GAMT: guanidino acetate methyl transferase, NOS: nitric oxide synthase, ORNT1: ornithine transporter 1, OTC: ornithine transcarbamylase, SLC6A8: solute carrier family 6 member 8 gene.

The urea cycle (Fig. 1), which is fully expressed only in the liver, forms urea starting from ammonia (NH_3_) derived from the nitrogen group of all amino acids [1]. It requires many enzymes and membrane transporters, any of which can be defective and cause a urea cycle disorder [2]. Ammonia escaping the urea cycle in periportal hepatocytes is conjugated with glutamate by glutamine synthase in perivenous hepatocytes to generate glutamine [3]. The accumulation of ammonia and glutamine in the brain leads to direct neuronal toxicity and brain edema [4], [5].

Urea cycle defects are treated with dietary protein restriction, nitrogen scavengers that bind glutamine or glycine, and supplements of citrulline or arginine (except in arginase deficiency). Arginine is one of the products of the urea cycle and can participate in the generation of nitric oxide or in the synthesis of creatine (Fig. 1). Creatine synthesis requires the action of two enzymes: arginine:glycine amidinotransferase (AGAT, OMIM 602360) and guanidinoacetate methyltransferase (GAMT, OMIM 601240) [6]. AGAT catalyzes the transfer of a guanidino group from arginine to glycine to form ornithine and guanidinoacetate (Fig. 1). GAMT catalyzes the transfer of a methyl group from S-adenosylmethionine to guanidinoacetate to form S-adenosylhomocysteine and creatine (Fig. 1).

Creatine then enters the brain and other tissues through the creatine transporter 1 (CT1, OMIM 300036) encoded by the *SLC6A8* gene. Cerebral creatine deficiency syndromes are inherited conditions caused by defects in either creatine biosynthesis or creatine transport that can cause delays in development, seizures, and movement disorders [6].

Previous studies found abnormalities in creatine metabolism in patients with urea cycle defects. Guanidinoacetate levels are elevated in the brain and plasma of patients with arginase deficiency [7], [8], [9], [10], [11]. Creatine levels are decreased in patients with ornithine transcarbamylase (OTC) and argininosuccinic acid synthase (ASS) deficiency whereas they are increased in argininosuccinic acid lyase (ASL) deficiency [12]. Guanidinoacetate is of particular interest since it can contribute to neurological damage in guanidinoacetate methyltransferase deficiency whereas creatine deficiency can directly impair brain activity [13]. It is unclear whether secondary alterations of creatine metabolism might contribute to some of the symptoms seen in patients with urea cycle defects.

Here we evaluate creatine and guanidinoacetate levels in patients with urea cycle disorders. Our data indicate that arginine levels directly correlate to the levels of guanidinoacetate and that creatine levels might be reduced in some patients with urea cycle disorders.

## 2 Materials and methods

### 2.1 Patients

This retrospective study was conducted according to protocols approved by the University of Utah Institutional Review Board. This study includes results from 207 plasma samples obtained from routine monitoring of 73 different patients (F = 37, M = 36) with urea cycle disorders (Table 1) on therapy, with an age range 0–56 years. Samples were collected during routine clinic visits, for which samples were collected about 3 h after the last meal or assumption of supplements. No samples were obtained during acute episodes of decompensation. All patients had normal serum creatinine levels. Some patients contributed more than one sample. To avoid bias, when multiple samples from the same patient were included in the study, the mean value for each patient was used in correlation and regression analysis. All patients received standard therapy for their urea cycle disorder, including low protein diet, citrulline supplements (100–200 mg/kg per day) in patients with OTC deficiency, arginine (100–200 mg/kg per day) in patients with ASS deficiency, and 150–400 mg/kg per day in patients with ASL deficiency. All patients were receiving sodium phenylbutyrate (200–500 mg/kg per day) as nitrogen scavenger, with none receiving sodium benzoate.

**Table 1:** Patients with urea cycle disorders and related samples analyzed in this study.

	OTC Deficiency	ASS Deficiency	ASL Deficiency	Arginase Deficiency	TOT
*Patients*	7	22	31	13	73
*Males*	3	12	15	6	36
*Females*	4	10	16	7	37
*Age range (y)*	0–22	0–34	0–56	0–25	
*Samples*	22	60	81	44	207

Abbreviation: OTC: ornithine transcarbamylase, ASS: argininosuccinate synthase, ASL: argininosuccinate lyase.

### 2.2 Measurement of GAA and creatine in patients with urea cycle disorders by UPLC-MS/MS

Plasma guanidinoacetate and creatine were measured by liquid chromatography tandem mass spectrometry (UPLC-MS/MS). Briefly, plasma samples (20 μL) were de-proteinized with methanol containing deuterated internal standards (d3-creatine and d2-GAA). After centrifugation, the extract was dried under nitrogen, derivatized with butanolic HCl, dried, reconstituted with water:acetonitrile (70:30) and injected into a UPLC-MS/MS system (Waters Acquity UPLC solvent/sample manager; Waters Quattro Premier™ tandem mass spectrometer). Creatine and GAA were chromatographically resolved by reverse phase chromatography (Acquity UPLC BEH C18, 1.7 μm, 2.1 × 100 mm, with a 0.2 μm in-line pre column filter) and then detected by tandem mass spectrometry, monitoring the characteristic transitions for creatine (188 > 90), GAA (174 > 101) and for the corresponding isotopically labeled internal standards (d_3_-Creatine:191 > 93; d_2_-GAA 176 > 103) [14], [15]. The analytical measurement range was 0.5–350 μmol/L for creatine and 0.25–25 μmol/L for GAA.

Plasma amino acids were measured in the same samples by HPLC-MS/MS using a modified aTraq™ Method Procedure [16].

### 2.3 Statistical analysis

Reference ranges for analytes were determined in normal controls using standard laboratory practices. Values for different parameters are reported in the text as mean ± SD (standard deviation). Comparisons between groups were performed using *t*-test nonparametric (Mann-Whitney test) and correlations among metabolites was assessed by linear regression analysis using SigmaPlot (Systat software)

## 3 Results

### 3.1 Plasma levels of guanidinoacetate (GAA) and creatine in patients with urea cycle defects

GAA and creatine concentrations were measured in 207 plasma samples from 73 patients with urea cycle disorders (Table 1). There was an almost equal distribution of males and females, ranging in age from newborn to 56 years of age.

**Fig. 2:**
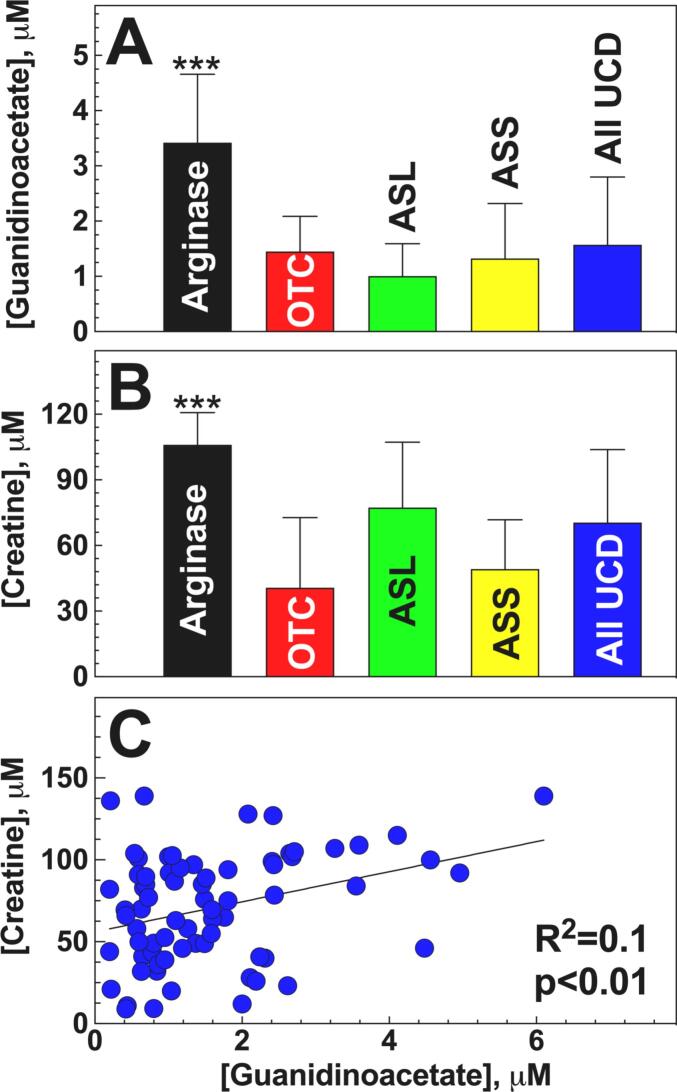
**Plasma levels of guanidinoacetate (A) and creatine (B) and their correlation (C) in patients with urea cycle defects.** A, B. Bars represent the mean ± SD of all samples from patients with the same diagnosis with UCD (urea cycle disorders). Comparisons between groups were performed using *t*-test using SigmaPlot (Systat Software) and are reported in the text as means ± SD. C. Linear regression analysis was used to determine correlations between creatine and guanidinoacetate with the parameters indicated in the graph. Abbreviations: Arginase = arginase deficiency (*n* = 13), ASL = Arginino-succinate lyase deficiency (*n* = 31), ASS = Argininosuccinate synthase deficiency (*n* = 22), OTC=Ornithine Transcarbamylase deficiency (*n* = 7), all UCD (all urea cycle defects, *n* = 73). *p < 0.01 versus other urea cycle disorders.

Levels of GAA were above the normal ranges (0.5–1.8 μM <11 y and 1.1–3.8 μM ≥11 y) in 34/44 samples of patients with arginase deficiency, with a mean value of 3.4 ± 1.2 μM, while GAA was above the normal range only in 5/22 samples of patients with OTC deficiency, 1/81 with ASL and 6/60 with ASS deficiency (Fig. 2A). GAA was below the normal range in 47/163 samples, these included 4/22 samples of patients with OTC, 24/81 with ASL, 19/60 with ASS deficiency, and none with arginase deficiency. The concentration of GAA measured in patients with arginase deficiency was significantly higher (*p* < 0.001 using *t*-test) than the concentration measured in other urea cycle disorders (OTC, ASL and ASS deficiency, Fig. 2A).

Mean creatine levels were in the normal range (9–90 μM <11 y and 37–117 μM ≥11 y) for patients with all urea cycle disorders (Fig. 2B). However, creatine was elevated in 12/44 samples of patients with arginase deficiency, 13/81 with ASL deficiency and 1/60 with ASS deficiency.

Creatine was overall in the normal range in OTC and ASS deficiency, and below the normal range in 1/22 samples of patients with OTC deficiency, 5/81 with ASL, 15/60 with ASS deficiency, and none with arginase deficiency. Patients with arginase deficiency had creatine levels significantly (*p* < 0.001) higher than patients with all other urea cycle disorders (Fig. 2B). Creatine levels positively correlated with GAA levels in plasma in all UCD patients (Fig. 2C). However, the correlation was not very strong (R^2^ = 0.1) suggesting that factors other than GAA concentration might affect the creatine pool in patients with UCD.

### 3.2 Correlation between plasma amino acids, guanidinoacetate, and creatine levels in patients with urea cycle defects

**Fig. 3:**
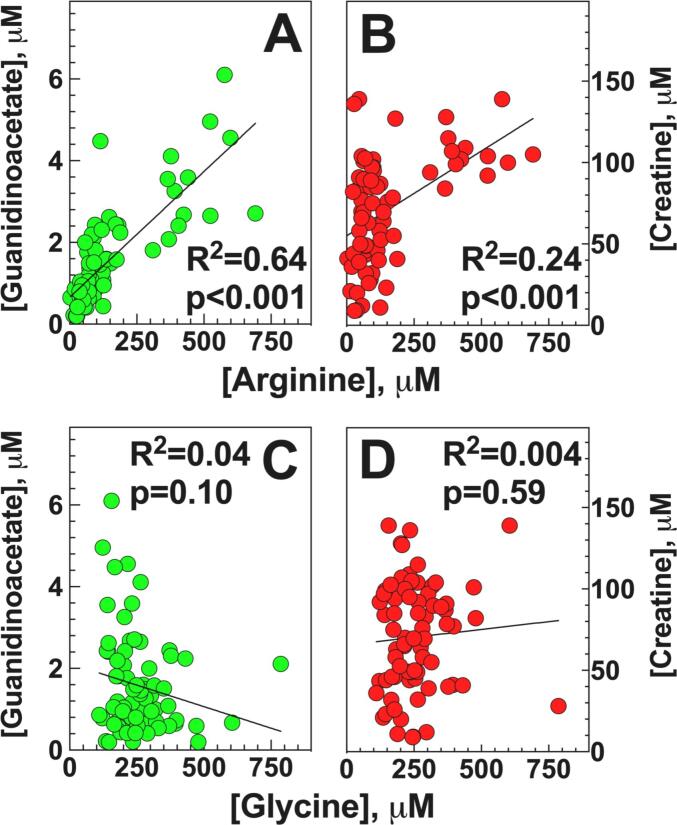
**Correlation of arginine (A, B) and glycine (C, D) with plasma levels of guanidinoacetate and creatine in patients with urea cycle defects.** Linear regression analysis was used to determine correlations between creatine and guanidinoacetate with different amino acids with the parameters observed indicated on the graphs.

Since arginine and glycine are the precursors of GAA and creatine, we analyzed the correlation between these two amino acids and GAA and creatine in patients with urea cycle disorders (Fig. 3). Both GAA and creatine positively correlated (*p* < 0.001) with levels of arginine (R^2^ = 0.63 for GAA and R^2^ = 0.23 for creatine, Fig. 3A and B), although the correlation with creatine was less robust. No significant correlation was observed between glycine and either GAA or creatine (Fig. 3C and D).

Patients with urea cycle disorders usually follow a protein-restricted diet that can result in reduced levels of amino acids in plasma [17]. In addition, phenylbutyrate, a commonly used therapy in urea cycle disorders, can decrease branched-chain amino acids [18]. We evaluated the correlation of each amino acid with GAA and creatine in patients with urea cycle disorders (Table 2).

**Table 2:**
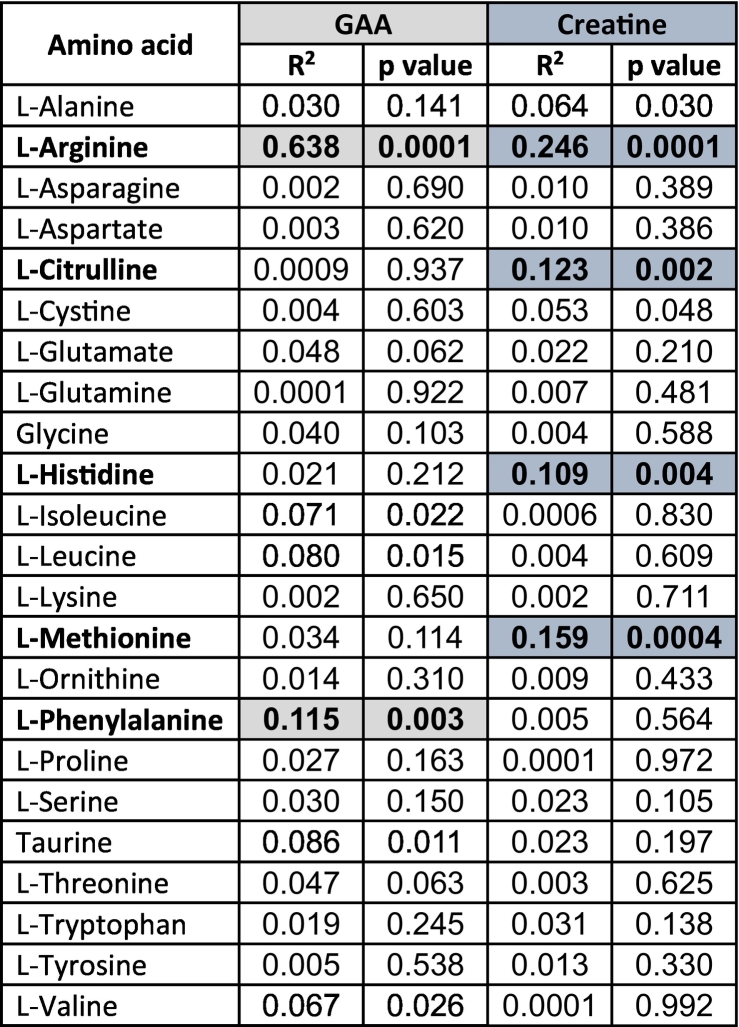
Correlation of individual plasma amino acids with guanidinoacetate (GAA) and creatine in patients with urea cycle disorders.

Amino acids whose correlation was highly significant (p < 0.01) are highlighted.

**Fig. 4:**
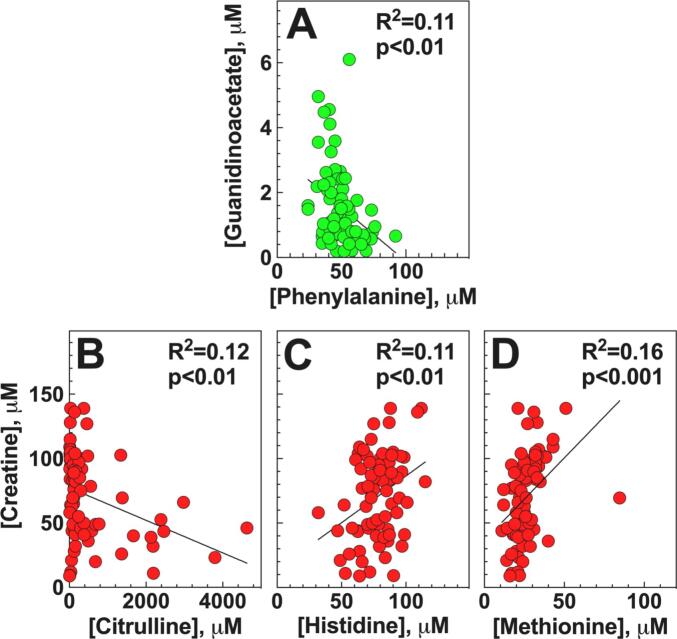
**Correlation of several amino acids with plasma levels of guanidinoacetate and creatine (A, B, C, D) in patients with urea cycle defects.** Linear regression analysis was used to determine correlations between guanidinoacetate with different amino acids with the parameters observed indicated on the graph.

Using p < 0.01 as a cutoff, GAA inversely correlated with phenylalanine (*p* < 0.01; R^2^ = 0.11; Fig. 4A). Creatine levels inversely correlated with citrulline (*p* < 0.01; R^2^ = 0.12, Fig. 4B), but directly correlated with histidine (p < 0.01; R^2^ = 0.11, Fig. 4C) and methionine (*p* < 0.001; R^2^ = 0.16, Fig. 4D). Ornithine, whose levels negatively correlate with GAA in patients with GAMT deficiency [15] did not corelate with either GAA or creatine levels in patients with urea cycle disorders (Table 2).

## 4 Discussion

The objective of this study was to identify variations of creatine metabolism in patients with urea cycle disorders. We evaluated GAA, creatine and amino acids concentration on 207 plasma samples from 73 patients with different UCD (Ornithine Transcarbamylase (OTC) deficiency, Argininosuccinate Synthase (ASS) deficiency, Argininosuccinate Lyase (ASL) deficiency and Arginase deficiency) (Table 1). Plasma concentration of GAA were above the normal range only in patients with arginase deficiency (Fig. 2A), being in the normal range in all other UCD. Some patients with arginase deficiency had levels of GAA overlapping with those of patients with guanidinoacetate methyltransferase deficiency, a defect of creatine biosynthesis [19]. However, creatine levels were normal or elevated in arginase deficiency, while they are reduced in guanidinoacetate methyltransferase deficiency at time of diagnosis [15].

Elevated GAA and other guanidino compounds have been previously reported in hyperargininemia/arginase deficiency [7], [8], [9], [10], [11], [20]. Patients with arginase deficiency differ from those with other urea cycle disorders since they can develop progressive spastic paraparesis even with mild or absent hyperammonemia [21]. For this reason, elevation of guanidino compounds, not seen in other urea cycle disorders, was proposed as a possible mechanism [20] of the phenotype of arginase deficiency. GAA was increased in the brain tissue of an adult, but not a child who died with arginase deficiency and, with other guanidino compounds, might play a role in generating neurological symptoms in arginase deficiency [7]. Extension of this study to all urea cycle disorders identified a very strong correlation between plasma levels of arginine and GAA (Fig. 3A). Arginine is a direct precursor of GAA (Fig. 1), whose synthesis is highly dependent on arginine levels [22]. By contrast, glycine, the other precursor of GAA, did not correlate significantly with either GAA or creatine (Fig. 3C and D). This is also seen in normal animals in which infusion of arginine, but not of glycine increases GAA levels [22]. This is in contrast to what is observed in GAMT deficiency, where the metabolic block prevents further conversion of GAA to creatine and glycine levels strongly correlate with GAA levels [15]. Nevertheless, the direct correlation between GAA and arginine in patients with all urea cycle disorders (Fig. 3A) suggests that reduction of arginine could reduce GAA and other guanidino metabolites in arginase deficiency.

GAA directly correlated with arginine but correlated negatively with phenylalanine (Table 2 and Fig. 4A). Low levels of this essential amino acid can be caused by decreased intake of protein, a standard therapy for patients with urea cycle disorders. Foods rich in protein (meat and dairy products) are the major source of creatine in our diet and protein restriction results in a secondary decrease in creatine intake. The inverse correlation between the concentration of the essential amino acid phenylalanine and GAA might reflect downregulation of arginine:glycine amidinotransferase, the enzyme that synthesizes GAA, because of increased protein and creatine intake [23].

Low-normal creatine levels have been reported in urea cycle disorders [12], with lower values in OTC and ASS deficiency compared with ASL deficiency. Creatine concentration was higher in patients with lysinuric protein intolerance (LPI) and ASL while on arginine supplementation, compared with patients with OTC and ASS deficiency [12], possibly as a consequence of low cellular arginine availability [24]. In our study, creatine levels were normal or elevated in arginase deficiency and normal in the other UCD (Fig. 2B). Plasma creatine levels were, overall, within the normal range in all UCD and directly correlated with arginine (Fig. 3B). This could be a consequence of the positive correlation of arginine with GAA (Fig. 3A), even though the correlation between GAA and creatine was not very strong (R^2^ = 0.07) (Fig. 3C), reflecting the contribution of other factors, such as dietary intake, to the creatine pool in UCD patients.

Several factors could contribute to the low GAA in some UCD patients. Patients with UCD are on a low-protein diet, omitting foods that are the major source of creatine [12]. Most urea cycle disorders impair arginine synthesis with secondary deficiency of intracellular arginine [25]. In theory, accumulation of ornithine could reduce GAA and creatine synthesis [26], which is the basis of ornithine therapy in GAMT deficiency [15]. However, our data (Table 2) show no inverse correlation between ornithine and GAA or creatine, making this possibility unlikely in classic urea cycle disorders. A negative effect of markedly elevated ornithine on creatine synthesis might however occur in hyperornithinemia-hyperammonemia-homocitrullinuria syndrome and ornithine amino transferase deficiency [12], [27], [28], conditions causing a marked elevation of plasma ornithine that were not investigated in our study.

Creatine directly correlated with histidine and methionine (Table 2 and Fig. 4). The GAMT enzyme that converts guanidinoacetate to creatine (Fig. 1) requires S-adenosylmethionine (SAM) as a methyl donor and consumes about 50% of all SAM-derived methyl groups [29]. Given the normal or increased abundance of GAA in patients with UCD, availability of methionine might become the rate limiting step in creatine biosynthesis explaining the direct correlation of this amino acid with creatine. Inconsistent availability of methionine (and S-adenosylmethionine) might also contribute to the relatively poor correlation between GAA and creatine (Fig. 2C). We have no explanations for the direct correlation between histidine and creatine, but histidine is involved in the synthesis of folic acid, an alternative methyl donor that might spare SAM from other reactions to increase availability for GAA methylation [30].

Creatine negatively correlated with citrulline (Fig. 4B), a precursor of arginine in a functional urea cycle and a product of arginine metabolism by nitric oxide synthase (Fig. 1). Citrulline levels are the highest in patients with citrullinemia type 1 (ASS deficiency) who have the lowest creatine levels [12] (Fig. 2B) and moderately increased in patients with argininosuccinic aciduria (ASL deficiency). The levels of creatine in these patients probably are directly related to the concentration of arginine within cells, being highest in arginase deficiency, followed by patients with ASL deficiency who routinely received in the past arginine supplements at high doses (600 mg/kg per day, [31]). Patients with ASS deficiency receive lower supplements of arginine and their administration is intermittent, possibly resulting in low intracellular levels between doses.

These variations in creatine metabolism might contribute to some of the clinical manifestations of patients with UCD. The minor decrease in creatine levels observed in some patients with UCD is probably not clinically significant, but the increase in guanidinoacetate (and likely of other guanidino compounds) in arginase deficiency might play a role in the neurological abnormalities peculiar to this urea cycle disorder.

## Funding

Dr. Filippo Ingoglia was supported in part by the 2019 Takeda Pharmaceuticals/ACMG Foundation Next Generation Fellowship Awards. The authors confirm independence from the sponsors; the content of the article has not been influenced by the sponsors.

## Declaration of Competing Interest

All authors state that they have no competing interests to declare. None of the authors accepted any reimbursements, fees or funds from any organization that may in any way gain or lose financially from the results of this study.
